# Transglutaminase 2 has opposing roles in the regulation of cellular functions as well as cell growth and death

**DOI:** 10.1038/cddis.2016.150

**Published:** 2016-06-02

**Authors:** H Tatsukawa, Y Furutani, K Hitomi, S Kojima

**Affiliations:** 1 Department of Basic Medicinal Sciences, Graduate School of Pharmaceutical Sciences, Nagoya University, Nagoya 464-8601, Japan; 2 Micro-Signaling Regulation Technology Unit, RIKEN Center for Life Science Technologies, 2-1 Hirosawa, Saitama 351-0198, Japan

## Abstract

Transglutaminase 2 (TG2) is primarily known as the most ubiquitously expressed member of the transglutaminase family with Ca^2+^-dependent protein crosslinking activity; however, this enzyme exhibits multiple additional functions through GTPase, cell adhesion, protein disulfide isomerase, kinase, and scaffold activities and is associated with cell growth, differentiation, and apoptosis. TG2 is found in the extracellular matrix, plasma membrane, cytosol, mitochondria, recycling endosomes, and nucleus, and its subcellular localization is an important determinant of its function. Depending upon the cell type and stimuli, TG2 changes its subcellular localization and biological activities, playing both anti- and pro-apoptotic roles. Increasing evidence indicates that the GTP-bound form of the enzyme (in its closed form) protects cells from apoptosis but that the transamidation activity of TG2 (in its open form) participates in both facilitating and inhibiting apoptosis. A difficulty in the study and understanding of this enigmatic protein is that opposing effects have been reported regarding its roles in the same physiological and/or pathological systems. These include neuroprotective or neurodegenerative effects, hepatic cell growth-promoting or hepatic cell death-inducing effects, exacerbating or having no effect on liver fibrosis, and anti- and pro-apoptotic effects on cancer cells. The reasons for these discrepancies have been ascribed to TG2's multifunctional activities, genetic variants, conformational changes induced by the immediate environment, and differences in the genetic background of the mice used in each of the experiments. In this article, we first report that TG2 has opposing roles like the protagonist in the novel Dr. Jekyll and Mr. Hyde, followed by a summary of the controversies reported, and finally discuss the possible reasons for these discrepancies.

## Facts

- Tissue transglutaminase (TG2) is a multifunctional enzyme that exhibits crosslinking, GTPase, cell adhesion, protein disulfide isomerase, kinase, and scaffold activities.
- By virtue of these multiple activities, TG2 is implicated in the regulation of cell growth, differentiation, and apoptosis.
- However, opposing effects have been reported regarding its roles even in the same physiological and pathological systems.
- To help understand these discrepancies, we summarize and discuss possible reasons for opposing effects in each case.

## Open Questions

- How can we measure the individual activity of multiple functions of TG2 *in vivo*?
- How are these multiple functions and genetic variants of TG2 regulated?
- How is TG2's substrate specificity (other than its sublocalization) determined?

**Figure 1:**
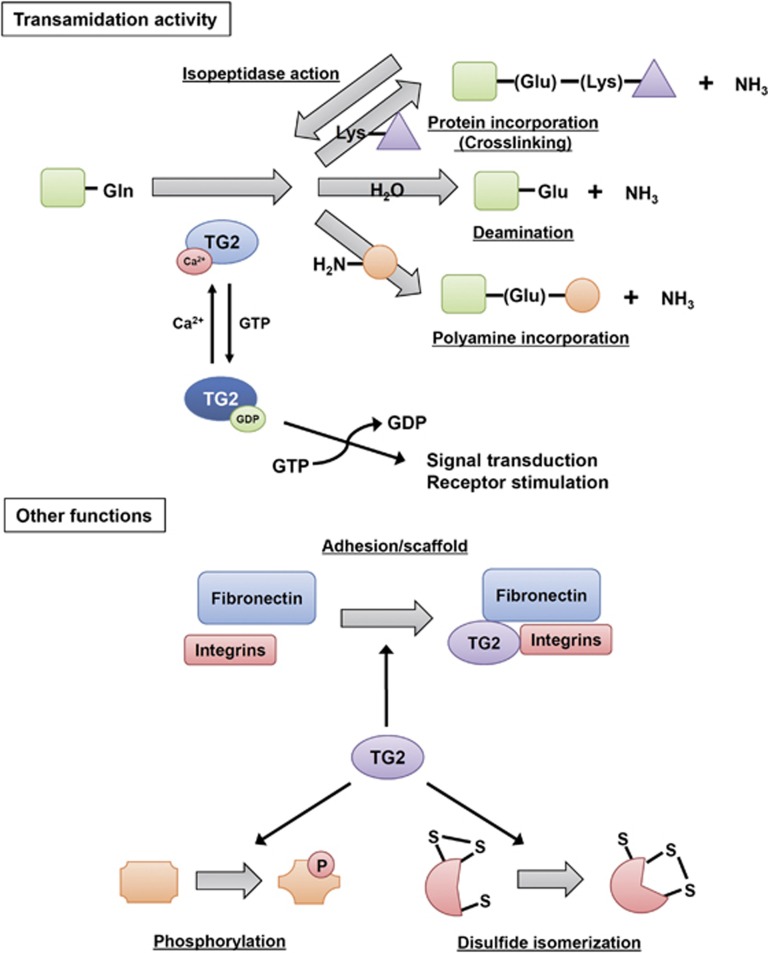
Diverse functions of TG2. TG2 regulates the post-translational modification of several proteins though several activities, including transamidation, GTPase, adhesion/scaffold, kinase, disulfide isomerase, and isopeptidase activities. Some functions of TG2 remain unclear. TG2 exerts different activities depending on the stimuli; these activities lead to several effects, including apoptosis, cell growth, and differentiation

Transglutaminase 2 (TG2) is a multifunctional enzyme and the most ubiquitously expressed member of the large TG family, a protein family of eight isozymes designated as blood coagulation factor XIII and TG1-7. TGs primarily deamidate *γ*-carboxamide groups of specific protein-bound glutamines while exchanging any primary amines, the ɛ-amino group of a lysine residue, or water, and form ammonia plus an N^ɛ^(*γ*-glutamyl)lysine crosslinking between glutamine and lysine residues or convert glutamine to glutamic acid.^1, 2, 3^ TG2 exerts additional enzymatic activities that do not require Ca^2+^;^1, 3^ that is, hydrolyzing ATP and GTP to mediate signal transduction through G-protein-coupled receptors,^4, 5^ protein disulfide isomerase and protein kinase,^6, 7^ interacting with several proteins as an adhesion or scaffold protein^8^ (Figure 1).

TG2 is predominantly found in the cytosol. TG2 also appears in the nucleus, mitochondria, plasma membrane, and extracellular matrix (ECM).^9^ In mammals, TG2 is widely present throughout the body including blood, extracellular spaces, and intracellular compartments of nearly all tissues, and induces tissue remodeling/wound healing and ECM assembly as well as cell growth, differentiation, and cell death.^10^ TG2 is thus involved in the pathogenesis/treatment of cancer, diabetes, neurodegeneration, fibrosis, inflammatory, and autoimmune disorders^2^ as well as liver diseases.^11, 12, 13^ For the general functions and structure of TG2, including topics on genetically engineered mouse models and inherited disorders, refer to well-written review articles and/or a book recently released.^2, 9, 14^

Opposing effects have been reported for the roles of TG2 in the same physiological and/or pathological systems due to differences in

1. The enzymatic and non-enzymatic activities or properties of TG2, that is, GTPase *versus* transamidation *versus* scaffold activities.^2, 15, 16^
2. Genetic variants including alternative splicing, that is, the short or truncated form (TG2-S) *versus* the long form (TG2-L)^17^ and single-nucleotide polymorphisms.^18^
3. The conformational structure (open *versus* closed forms), which is affected by immediate cellular and tissue environments.^2, 15^
4. The genetic background of mice.^2, 13^

The aim of this review is to summarize recently obtained knowledge of how TG2 plays Dr. Jekyll and Mr. Hyde (opposing roles) in the regulation of cell growth and death.

## Multifunctional Activities of TG2 and Its Regulation

### GTPase

Intracellular TG2 in its closed form acts as a Ca^2+^-independent GTPase in normal cells when the intracellular Ca^2+^ concentration is as low as 10–20 nM, participating in the transmembrane signaling of phospholipase Cδ as a component of *α*_1_-adrenergic receptor complexes and supporting the growth of hepatic cells.^16, 19, 20^ GTP binding with TG2 sustains the closed form and prevents its conformational change to the open form; the emergence of crosslinking activity is thus a result of binding to Ca^2+^, whereas the depletion of GTP causes conformational changes in the open form. In this scenario, the emergence of crosslinking activity induces apoptosis in islet *β*-cells^21^ and other cell types.^22^

### Transamidase

When cells are injured (or receive certain stimuli) and the intracellular Ca^2+^ concentration increases to more than 700–800 nM, TG2 markedly alters its structure and transforms into an open form that exerts crosslinking activity.^16, 23^ In the specific conditions such as tissue regeneration and remodeling, TG2 crosskinks ECM proteins, however, despite a high extracellular Ca^2+^ concentration (in the mM range) outside of cells, TG2 usually does not exert transamidation but instead mediates multiple and complex regulatory interactions, particularly in the regulation of cell-ECM interactions and outside-in signaling via transmembrane receptors, indicating that Ca^2+^ and/or GTP concentrations are not the sole determinants of transamidation activity.

### Integrin–fibronectin interaction

TG2 has been identified as an important extracellular crosslinking enzyme involved in ECM turnover.^24, 25, 26, 27^ In contrast, TG2 is secreted into ECM and forms a hetero complex with its high-affinity binding partner fibronectin (FN) through its N-terminal 42-kD fragment in a crosslinking activity-independent manner.^28, 29^ The TG–FN complex promotes FN fibril deposition and RGD-independent cell adhesion via syndecan-4/2 and *α*5*β*1 integrin co-signaling,^29^ and sustains cell survival in osteoblasts,^30^ bone marrow-derived mesenchymal stem cells,^31^ and many tumor cells.^32, 33^

### Miscellaneous functions

Protein disulfide isomerase (PDI) activity^6^ has been implicated in mitochondrial-dependent apoptosis,^34^ whereas TG2 has been reported to exert an intrinsic serine/threonine kinase activity and to phosphorylate insulin-like growth factor (IGF)-binding protein-3 (IGFBP3),^7^ p53 tumor suppressor protein,^35^ H1-4 histones,^36^ and Rb protein.^37^ Moreover, TG2 affects the hypusination of eukaryotic initiation factor 5A (eIF5a) in BALB/c 3T3 cells;^38^ this action may be related to the fact that eIF5a serves as a binding protein and/or substrate for TG2.^39, 40^

### Regulation

The gene expression related to TG2 is modulated by endoplasmic reticulum (ER) stress,^11, 41^ tissue remodeling,^42^ inflammation,^2, 43^ viral infection,^44^ and apoptotic signals,^45, 46^ as well as cancers^2, 47, 48, 49^ and mediated by soluble factors such as transforming growth factor (TGF)-*β*/bone morphogenetic protein 4,^50^ interleukin (IL)-1,^51^ IL-6,^52^ tumor necrosis factor (TNF)-*α*,^53^ and epidermal growth factor (EGF)^47^ (Table 1). Retinoic acid (RA) induces TG2 expression;^54, 55^ in acute promyelocytic leukemia treated with RA, TG2 has an important role in the neutrophil–granulocyte differentiation and gene expression of neutrophil's cellular functions in addition to related adhesive, migratory, and phagocytic capacities.^56^ The transcriptional activation of the TG2 gene, *TGM2*, is mediated by nuclear factor-kappa B (NF-*κ*B),^41^ RA receptor (RAR)/retinoid X receptor, liver X receptor/RAR, and Sp1.^55^

Two examples of a positive feedback loop are known to act in the regulation of TG2 expression. TG2 is indispensable for latent TGF-*β* activation in many tissues,^57, 58, 59, 60, 61, 62, 63^ and the generation of TGF-*β* may stimulate TG2 gene expression. TG2 activates NF-*κ*B via the depletion of inhibitor of *κ*B (I-*κ*B)*α* via polymerization in the absence of I-*κ*B*α* kinase activation. This activity results in the dissociation of NF-*κ*B and its translocation to the nucleus, where it may upregulate TG2 in cancer cells.^64, 65, 66^

Ca^2+^ and nucleotides (GTP/ATP), respectively, act as the activator and suppressors of the transamidase activity of TG2. Nitric oxide (NO) is also a potent inhibitor by promoting S-nitrosylation on the active site C277.^67^ The decreased S-nitrosylation of TG2 contributes to age-related increases in vascular stiffness.^68^ Micro-RNA (miR-19) directly downregulates TG2 expression and enhances the invasion of colorectal cancer cells.^18^ Finally, the SUMOylation of TG2 enhances its protein levels and activity by blocking the ubiquitination of (and thus stabilizing) TG2 protein.^69^

## Opposing Roles of TG2 *In Vitro*

The accumulation of TG2 in various cell and tissue types undergoing apoptosis has been demonstrated;^70, 71^ elevated TG2 activity is correlated with enhanced apoptosis in a neuroblastoma cell line,^72^ whereas treatment with antisense against TG2 reduces apoptosis.^73^ When neuroblastoma cells are transfected with plasmid overexpressing TG2 but not mutated TG2 lacking crosslinking activity and then treated with staurosporine or osmotic stress, an increased crosslinking activity accompanies the activation of caspase-3 and apoptotic nuclear changes.^74^ In mitochondria, interaction with Bax through TG2's BH3 domain plus the predicted crosslinking of Bax causes mitochondrial depolarization, the release of cytochrome *c*, and cell death.^45^ Nuclear TG2 causes caspase-independent cell death via the crosslinking and inactivation of the general transcription factor Sp1, resulting in the reduced expression of growth factor receptors, such as c-Met and EGF receptors, that are essential for cell survival.^12, 75^ In contrast, numerous reports have found a protective effect of TG2 in cell death induced by TNF-*α*,^76^ RA,^77^ and stressors such as thapsigargin, hyperosmotic stress, and oxygen/glucose deprivation.^78^

Below are examples in which TG2 has Dr. Jekyll and Mr. Hyde (opposing functions; Tables 2,3,4,5,6).

## Neuroprotective *Versus* Neurodegenerative Roles by Modulating HIF1 and Huntingtin Functions, Respectively

Increased activity and expression of TG2 are observed in the ischemic hippocampus after reperfusion (Table 2).^79, 80^ TG2 expression is induced by oxidative stress, and the induction of inducible NO synthase and NO production, which contribute to neurodegeneration, are closely associated with TG2 expression in the lipopolysaccharide-stimulated activation of astrocytes.^81^ The infarction volume was smaller in both TG2 knockout mice and mice treated with cystamine than in control mice.^82^ In contrast, both SH-SY5Y human neuroblastoma cells^83^ and a permanent middle cerebral artery ligation stroke model^84^ demonstrated that hypoxic conditions increased nuclear TG2, TG2 binding to HIF1*β* independently of transamidase activity, and the prevention of the upregulation of pro-apoptotic Bnip3^85^ and Noxa,^86^ thereby protecting neuronal cells from hypoxia-induced death in ischemia and stroke.^83, 84^

Increased TG2 levels and/or activity, especially the nvolvement of nuclear TG2, have been observed in many neurodegenerative diseases such as Alzheimer's disease (AD), Huntington's disease (HD), and Parkinson's disease (PD).^87^ AD is characterized by the formation of extracellular neurotoxic aggregates consisting of amyloid-*β* protein or intracellular neurotoxic aggregates consisting of hyperphosphorylated tau. TG2 mediates the crosslinking of both amyloid-*β* and tau *in vitro*^88, 89^ and the polyamination of the tau protein, resulting in an increased resistance of tau to proteolytic degradation by calpain. This change leads to higher levels of non-degradable tau within the neuron,^90^ which indicates that TG2 may accelerate the aggregation process of amyloid-*β* and tau in AD patients. Furthermore, the enhanced expression of a short form of TG2 (sTG2), an alternatively spliced transcript lacking GTPase activity that maintains poor crosslinking activity and thus has a pro-apoptotic property, has been reported in AD patients,^22, 91, 92^ whereas TG2 is not a biochemical marker for AD disease because no colocalization of TG2 with tau or amyloid-*β* deposits is found in neocortex sections.^93^

In the frontal cortex of postmortem HD brain tissues, 99% colocalization is observed between ɛ-(*γ*-glutamyl)lysine crosslinks and huntingtin aggregates in the nucleus,^94^ indicating an involvement of nuclear TG2 in HD. Furthermore, the TG inhibitors cystamine and monodansylcadaverine partially suppress aggregate formation and apoptosis in cells expressing truncated dentatorubral-pallidoluysian atrophy protein with an expanded polyglutamine stretch.^95^ Although, (i) the *in vivo* ablation of the TG2 gene ameliorates HD symptoms and leads to unaltered or even increased numbers of neuronal intranuclear inclusions,^96, 97^ which has recently been ascribed at least in part to a loss of TG2's regulatory effect on autophagy;^98^ (ii) cystamine inhibits not only TG2 but also caspase-3;^99^ (iii) neuronal intranuclear inclusions are likely to be formed at a late stage of aggregation and do not directly affect the progression of pathogenesis;^100^ and (iv) the genetic deletion or enzymatic inhibition of TG2 alleviates the degenerative process and improves survival as well as life span,^97, 101^ isolated polyglutamine forms aggregates through a *β*-sheet-based nucleation mechanism, indicating a causative role for nuclear TG2 in HD pathogenesis via mechanisms other than the formation of neuronal intranuclear inclusions.^102^

Transcription dysregulation and impaired energy homeostasis have important roles. The introduction of ZDON, a peptide inhibitor of TG2, ameliorates HD symptoms.^101, 103^ The mutant huntingtin binds to and inactivates other polyglutamine-enriched proteins such as transcription factors, including the general transcription factor Sp1 or its coactivator TAFII130,^104, 105, 106^ which may repress the Sp1-dependent transcription of BDNF,^107^ dopamine D2 receptor,^104, 105^ preproenkephalin,^104^ the mitochondrial proteins PGC-1*α,* and cytochrome c.^108, 109^ These results imply that although nuclear TG2 is not essential for inducing HD, it may be an important factor in exaggerating HD symptoms through the transcriptional dysregulation of these survival factors and key metabolic genes. Defective nuclear actin remodeling causes faster cell death in correlation with disease progression.^110^ Cofilin is an actin binding protein that is required for actin treadmilling.^111^ The formation of the dynamic cytoskeleton, referred to as 'actin-cofilin rods', is a self-protecting reaction against cellular stress and is sustained by an association with normal huntingtin upon its release from the ER and localization to the nucleus under stress conditions. In response to cellular stress, delayed and aberrant actin-cofilin rods are formed in neurodegenerative disease by (i) mutant huntingtin via the impairment of the normal function of huntingtin and (ii) an excessive crosslinking of actin-cofilin complexes by stress-activated nuclear TG2.^110^ As stated above, TG2 binds FN through its N-terminal domain and interacts with many other scaffold proteins such as integrins and lamins A and C.^28, 112, 113^ In PD patients' brains, TG2 expression is increased in the substantia nigra, and TG2-catalyzed crosslinking has been shown to colocalize with *α*-synuclein, a substrate for TG2 *in vivo*,^114^ in dementia with Lewy bodies.^115^

## Hepatic Protective *Versus* Insult Roles Through Sp1 Crosslinking

TG2 crosslinking activity significantly increases with carbon tetrachloride (CCl_4_) or ethanol-induced liver injury in rats and in acute human liver injury (Table 3).^116, 117, 118, 119, 120^ Cystamine and garlic extract prevent CCl_4_-induced liver injury and fibrosis via the inhibition of TG2.^121^ Sp1 is a general transcription factor that is rich in lysine and glutamine residues,^122^ thereby serving as a good substrate for TG2 both *in vitro* and *in vivo*.^12^ An upregulation of Sp1's transcriptional activation activity upon crosslinking with nuclear TG2 in human 293T cells likely occurred because Sp1 exerts higher transcriptional activation activity as a dimer or trimer,^123, 124^ whereas in alcohol or free fatty acid (FFA)-treated hepatic cells, highly crosslinked, oligomerized Sp1 loses its transcriptional activation activity. A defect in Sp1 activity causes the decreased expression of *c-Met*, the major receptor for hepatocyte growth factor, leading to caspase-independent hepatic cell death in culture systems and animal models as well as in patients with both alcoholic steatohepatitis (ASH) and non-ASH (NASH).^11, 12, 75^ FFAs increase ER stress, NF-*κ*B activation, and nuclear TG2 through a pancreatic ER kinase (PERK)-dependent pathway, whereas ethanol-induced nuclear TG2 is dependent at least in part on retinoid signaling.^11^ RA enhances the transcription of the *TG2* gene via GC box motifs in its promoter region through a physical interaction between newly synthesized RARs and preexisting Sp1.^55^ Therefore, the crosslinking and silencing of Sp1 by TG2 may be a feedback mechanism to control the excessive expression of TG2 and prevent it from playing Mr. Hyde.

Furthermore, TG2 contributes to the clearance of apoptotic cells by promoting monocyte infiltration via the dimerization of the monocyte chemotactic factor S19^125^ followed by macrophage engulfment via integrin *β*3,^126^ leading to inflammation suppression. In addition, TG2 contributes to wound repair and tissue stabilization via the crosslinking of various intracellular and extracellular proteins at inflammation sites.^42^ A peptide with anti-TG2 activity decreases lung inflammation accompanied by reduced neutrophil infiltration and cytokines expression.^127^

However, TG2 has been reported to be anti-apoptotic, and the effect is linked to both its GTP binding and crosslinking activity.^15, 20^ TG2-null mice do not exhibit an obvious hepatic phenotype defect but do exhibit an impaired clearance of apoptotic cells by phagocytosis under stress conditions and experience a more severe liver injury after CCl_4_ or Fas administration.^20, 128^ This discrepancy can be explained as follows. Sarang *et al.*^20^ reported that TG2 protects against high-dose (1 *μ*g/g body weight) Jo2 (anti-Fas antibody)-induced liver injury; TG2^−/−^ mice were more sensitive to Jo2-mediated necrosis than TG2^+/+^ animals. In those studies, Sarang *et al.*^20^ used FVB mice as wild-type controls. We used wild-type littermates from heterozygous TG2^+/−^ crosses and applied a high dose of Jo2 (used by Sarang *et al.*^20^); this treatment resulted in massive hepatic necrosis both in TG2^+/+^ and TG2^−/−^ mice. Thus, the varying genetic backgrounds of the TG2^+/+^ *versus* TG2^−/−^ mice used by Sarang *et al.*^20^ might have contributed to the discrepancy between their findings and ours. However, we reproduced the protective effect of TG2 silencing on hepatic injury with a low dose (0.1 *μ*g/g body weight) of Jo2 using the exact same TG2^−/−^ mouse line used by Sarang *et al.*^20^ Thus, TG2 may promote the hepatic apoptosis caused by relatively low doses (but not higher doses) of Jo2.

Using the same TG2^−/−^ mouse line used by Sarang *et al.*,^20^ Nardacci *et al.*^128^ reported that TG2 is protective in CCl_4_-mediated liver injury and speculated that the observed increase in TG2 expression during the initial stages of liver fibrosis in HCV-infected patients may protect against liver injury. However, another explanation may be that high levels of TG2 contribute to liver injury in these patients; as we have shown in an animal model,^12^ TG2 inhibitors may be a useful treatment for the prevention of hepatic apoptosis.

## Fibrogenic *Versus* No or anti-Fibrogenic Functions of TG2

In injured liver cells, TG2 transforms into a crosslinking enzyme (open form) and contributes to the wound healing process and fibrosis (Table 3). First, the crosslinking reaction results in the formation of an N^ɛ^(*γ*-glutamyl)lysine isopeptide bond, which is one important step in the maturation or stabilization of ECMs (such as collagens) in the extracellular space, exacerbating hepatic fibrosis.^117, 129^ The N^ɛ^(*γ*-glutamyl)lysine crosslink, which is undetectable in normal liver tissue, is present extracellularly in the fibrotic livers of patients with a variety of chronic liver diseases, primarily in inflammatory areas in which an intense remodeling is occurring.^117^ Second, this crosslinking ability of TG2 appears to have a crucial role in the fixation and activation of TGF-*β*,^57^ the most fibrogenic cytokine.^130^ TGF-*β*1 is released in a latent form (~300 kD) and converted to an active form of 25 kD. Enhanced TG2 activity is required in many tissues for this activation of TGF-*β* via its crosslinking of large latent complexes to the cell surface or to FN and other ECM components through a latent TGF-*β*-binding protein portion.^57, 61, 62, 131, 132^ In addition to the liver, similar fibrogenic roles via the generation of TGF-*β* have been reported in articular cartilage,^60^ kidney,^61^ lung,^59^ and pancreas.^133^

Third, in *in vivo* models of hepatic apoptosis and in ASH patients, Sp1 is crosslinked, oligomerized, and inactivated by nuclear TG2, leading to the activation of a caspase-independent apoptotic process resulting from the reduced expression of *c-Met*.^12^ The TG2-induced decrease in *c-Met* may be involved in the impaired hepatocyte regeneration observed in patients with alcoholic liver diseases.^13, 134, 135^ Furthermore, Giebeler *et al.*^136^ reported that the downregulation of *c-Met* is associated with liver fibrosis, indicating a novel apoptotic axis accompanied by liver fibrosis, namely, a nuclear TG2/crosslinked Sp1/decline in *c-Met*. Supporting this hypothesis, the nuclear accumulation of TG2 and crosslinking of Sp1 are observed in the fibrotic area of patients with ASH.^137^

In summary, increased TG2 activity is associated with ECM production and TGF-*β*, as shown after chronic CCl_4_ intoxication in rats^118^ directly by stabilizing the ECM in an insoluble form and indirectly by promoting the generation of active TGF-*β*, which strongly enhances hepatic fibrogenesis by increasing ECM production. The reaction is Ca^2+^ dependent and is classically reported to be the biochemical basis for TG2 involvement in hepatic fibrosis.^129^ Therefore, amine substrates, such as putrescine and cystamine (competitive inhibitors of the crosslinking activity of the enzyme), have been shown to be protective in ethanol-induced liver injury as well as liver fibrosis induced by CCl_4_.^138, 139, 140^

In contrast, Popov *et al.*^141^ demonstrated no change in the extent of liver fibrosis after the treatment of TG2^−/−^ mice with CCl_4_ or thioacetamide. Again, potential explanations for this discrepancy include differences in mouse background, the method used for *TG2* gene targeting, and broad biological and non-specific activities of TG inhibitors.

## Anti- *Versus* Pro-Apoptotic Roles of TG2 in Cancer Cells Partially Through the Regulation of Rb and E2F1 Activities

Multiple studies have shown elevated TG2 expression in many types of cancer cells^9^ to be associated with increased drug resistance, metastasis, and poor patient survival (Table 4).^33, 142^ For example, an analysis of more than 30,000 genes from tumor samples revealed that *TG2* is a highly expressed gene in pancreatic adenocarcinoma.^143^ An important property of the highly malignant tumor cells is their ability to survive in hostile host environments as they passes through the lymphatic system or the bloodstream in their attempt to colonize distant sites. TG2 stabilized contact points of tumor cells with the subendothelial matrix in free-floating melanoma cells isolated from arterioles.^144^ In contrast, the downregulation of TG2 expression in melanoma cancer cells promoted their ability to metastasize.^145^ The ectopic expression of TG2 in a highly malignant hamster fibrosarcoma cell line significantly reduces tumor incidence despite the fact that TG2-transfected clones exhibit no significant differences in growth rates, cell morphology, or levels of spontaneous apoptosis *in vitro*.^146^ This finding indicates a suppressive effect of TG2 on tumor growth and confirms the importance of TG2 in the phenotypic changes associated with cancer. Similar nuclear TG2-mediated cell death (as observed in ASH and NASH) has been found in the chemoprevention of cancer. Acyclic retinoid, a synthetic retinoid, stimulates the nuclear localization and activation of TG2,^147^ resulting in the crosslinking and inactivation of Sp1, thereby causing cell death in hepatocellular carcinoma cell lines through the downregulation of EGF receptors.^75^ In contrast, TG2 enhances EGF receptor expression in glioblastomas and then induces cell transformation.^148^ Therefore, a precise understanding of the expression and function of TG2 in the context of cancer stages and types is important for the implementation of TG2-based interventions to disrupt malignant invasion, growth, and survival.

In U937 human leukemic monocyte lymphoma cells undergoing apoptosis, nuclear TG2 polymerizes Rb protein, which culminates in the loss of anti-apoptotic action by Rb protein due to its interaction with E2F1 and the prevention of Rb degradation, which accelerates the degradation of E2F1 and leads to cell growth arrest/apoptosis.^149^ In contrast, in some cell lines (NIH3T3 mouse embryonic fibroblast cells, HL-60 human promyelocytic leukemia cells, and mouse embryonic lung fibroblasts) treated with RA or *N*-(4-hydroxyphenyl)retinamide, TG2 prolongs the anti-apoptotic action of Rb protein,^150^ protecting it from degradation by caspase-7 due to transamidation and/or the GTP-binding activities instead of undergoing polymerization. This conclusion has been drawn from the finding that monodansylcadaverine, a transamidation inhibitor, blocks the protective effect, whereas a transamidation-defective [C277V] TG2 mutant also exerts a protective effect.^150^ The latter is corroborated in HEK293 human embryonic kidney cells in which nuclear localization of the [C277S] TG2 mutant attenuates apoptosis due to its GTP binding or scaffold protein characteristics upon complexing with Rb protein.^151^ Furthermore, the phosphorylation of the Rb protein at Ser780 by TG2 destabilizes the Rb-E2F1 complex, ameliorating apoptosis in MCF-7 human breast carcinoma cells, which is further stimulated by the phosphorylation of TG2 with protein kinase A and abrogated by high Ca^2+^ concentrations.^37^ These observations indicate that in general, although the outcome differs depending on cell types and treatments, nuclear TG2 heavily polymerizes Rb protein under extremely high Ca^2+^ concentrations, and low ATP concentrations, resulting in apoptosis through accelerating E2F1 degradation; otherwise, nuclear TG2 stabilizes Rb protein through the phosphorylation of Ser780 and the transamidation of certain glutamine residue(s) (but not through crosslinking) and preventing Rb degradation as a scaffold protein. This behavior indicates that intranuclear Ca^2+^ and GTP concentrations are important in determining whether TG2 acts as Dr. Jekyll or Mr. Hyde (opposing functions).^16^

## Diabetes

TG2 is involved in the membrane-mediated events required for glucose-stimulated insulin release from pancreatic *β* cells (Table 5).^152, 153, 154^ Vitamin A also induces insulin secretion from islets via TG2,^155^ and steroid hormone-induced TG2 facilitates the crosslinking of IGFBP1 and increases IGF-I actions.^156^ As a controversial effect, inhibitors of TG2, such as monodansylcadaverine and glycine methylester, do not prevent insulin secretion induced by either cAMP or phorbol ester at basal levels (10 nM) of Ca^2+^; however, these inhibitors prevent insulin secretion induced by Ca^2+^.^157^ These data indicate that the transamidation activity of TG2 has a critical role in insulin secretion.

TG2^−/−^ mice have impaired glucose-stimulated insulin secretion and show glucose intolerance after intraperitoneal glucose loading.^158^ The TG2^−/−^ mouse phenotype resembles that of maturity-onset diabetes in young patients who have several missense mutations located near the catalytic site of TG2 and impaired transamidation activity.^158, 159^ However, TG2-disrupted mice and the constitutive transamidation active form of TG2-expressing mice show no significant differences in responses to a glucose or insulin challenge, suggesting that glucose homeostasis is TG2 independent.^160^

## Blood Vessel Formation

TG2 has a positive role in the formation and stability of blood vessels (Table 6).^161, 162^ Coeliac disease-specific autoantibodies targeting TG2 disturb several steps of angiogenesis, including endothelial sprouting, the migration of both endothelial, and vascular mesenchymal cells, ECM degradation, the organization of the actin cytoskeleton in capillary cell types, alterations of cell-ECM interactions to thereby affect endothelial cell adhesion, polarization, and motility.^163, 164, 165^ At the endothelium, TG2 co-localizes with integrin and has an important role in cell spreading and adhesion.^113, 166^ Moreover, TG2 is co-localized with endostatin in the ECM secreted by endothelial cells under hypoxia, which stimulates angiogenesis and tumorigenesis.^167, 168^ TG2 inhibition leads to a reduction in FN deposition and matrix-bound VEGFA in HUVECs; TG2 thus positively regulates angiogenesis in a VEGF-dependent manner.^169^ Microarray data show that TG2 is specifically upregulated by turbulent shear stress in coronary artery endothelial cells.^170^ Given the important role of shear stress in the development of atherosclerosis, we assert that TG2 relates the progression of this disease. In an opposite effect, matrix changes induced by TG2 lead to the inhibition of angiogenesis and tumor growth,^171^ indicating that endothelial TG2 primarily promotes blood vessel formation via enhanced stabilization and migration of neovascularization; however, a part of extracellular TG2 reduces organized vasculature via the excess stabilization and accumulation of ECM following their crosslinking. In cultured endothelial cells, TG2 suppressed cell migration via the formation of TGF-*β*.^57^ In vascular smooth muscle cells, TG2 is involved in the all-*trans* RA-mediated apoptosis and reduction of neointimal mass in balloon-injured blood vessels.^54^ More recently, TG2 was shown to block extracellular VEGF's binding to heparan sulfate proteoglycans and inhibit an early stage of angiogenesis.^172^

Thus, the effect of TG2 on angiogenesis appears to differ depending on its stage and TG2's subcellular localization.

## Conclusions and Prospects

The crosslinking activity of TG2 appears to represent the dominant function of TG2 during apoptotic cell death accompanied by an elevation of intracellular Ca^2+^. In addition to crosslinking activity, at normal intracellular Ca^2+^ concentrations, the diverse cellular functions of TG2 appear to be attributed to G_h_, PDI, and kinase activities. These various biochemical activities of TG2 are differentially regulated depending on its subcellular localization; they also determine whether TG2 has Dr. Jekyll or Mr. Hyde. In the outer membrane, TG2 is released from cells by an unknown mechanism and constitutively exhibits crosslinking activity for ECM and anchoring protein. In the intracellular membrane and cytosol, TG2 typically adopts a closed conformation with the binding of GTP but not that of Ca^2+^, which contributes to intracellular Ca^2+^ homeostasis, cell proliferation, and other actions. Both growth-stimulating (anti-apoptotic) and pro-apoptotic functions of TG2 may depend on the cell type, the type of death stimuli, the intracellular localization of the enzyme and which of its activities are switched on.^15^

Thus, the use of an existing inhibitor, such as cystamine, is not effective for the regulation of TG2-related pathogenesis. It is important to develop specific regulatory compounds against crosslinking, GTPase, FN-binding, PDI, the phosphorylation activities of TG2, and its cofactors. To control cell death and survival, we are now establishing and screening inhibitors that regulate the nuclear localization of TG2.

## Figures and Tables

**Table 1:** List of factors reported to regulate TG2 expression and activity

**Name**	**Cell types**	**Phenotypes**
Ethanol	Hepatocytes	Hepatic injury^12^
Retinoid (retinoic acid) and retinoid receptors	Most cell types, including hepatocytes, hepatocellular carcinoma cells, endothelial cells, leukemia cells, and neuronal cells	Apoptosis,^54, 75^ differentiation,^56, 173^ chemoresistance^47^
Free fatty acids	Hepatocytes	Hepatic injury, lipid accumulation^11^
IL-1	Meniscal cells	Inflammation^51^
IL-6	Hepatoblastoma cells	Inflammation^52^
NF-*κ*B	Hepatocytes, breast cancer cells, neuronal cells	Hepatic injury,^11, 118^ hepatic fibrogenesis,^118^ chemoresistance^41, 49^
TNF-*α*	Hepatocytes	Hepatic fibrogenesis^53^
TGF-*β*/bone morphogenic protein 4	Epithelial-like cells from mink lung, preosteoblastic cells	Not examined
Nitric oxide	Endothelial cells, neuronal cells	Vascular stiffness,^68^ neurodegenerative disease^81^
Protein inhibitor of activated STAT (PIASy), E3 SUMO-protein ligase	Bronchial epithelial cells with cystic fibrosis, lung carcinoma	Autophagy^174^
miR-19	Colorectal cancer	Downregulation of TG2 leads to enhanced invasion and metastasis^18^

**Table 2:** Examples of opposing functions of TG2 in the brain

**Brain**	**Reports**	**Possible causation**	**References**
Protective effect	Hypoxia-induced cell death in ischemic stroke Infarct volume in human TG2-expressing mice was reduced.	Cell proliferation of GTPase activity is enhanced by neuronal TG2 overexpression and nuclear localization.	^84^
Degenerative effect	Huntington's disease in R6/1 transgenic mice Polyglutamine disease in spinocerebellar ataxia-1 transgenic mice Crosslinking of huntingtin with expanded polyglutamine, *β*-amyloid, tau, and *α*-synuclein in Huntington's, Alzheimer's, and Parkinson's diseases	Genetic knockout or inhibitor of TG2 attenuates neurodegenerative disease regardless of whether TG2 is involved in the formation of polyglutamine. The open conformation of TG2 is toxic in cytosol, whereas the closed conformation of TG2 promotes survival in the nucleus.	^96, 97, 175, 176^ ^177^ ^88, 114, 175, 178, 179^ ^82, 180^
	Cell death in ischemic stroke Infarct volume in TG2^−/−^ mice was reduced.		
	Short form of TG2	The short form of TG2 induces cell damage and death	^91^

**Table 3:** Examples of opposing functions of TG2 in the liver

**Liver**	**Reports**	**Possible causation**	**References**
Protective effect	Activation of Sp1 via its dimerization	Difference in cell types	^123^
	CCl_4_ and Fas-induced liver injury		^20, 128^
Promotive effect	CCl_4_-induced liver injury and fibrosis	Different animal backgrounds	^121^
	Liver damage and injury in ASH/alcohol, NASH/FFAs		^11, 12, 136, 137^
	Liver damage in NZB/W F1 mice (a well-known lupus-prone strain)		^139^
No effect	CCl_4_- or thioacetamide-induced liver fibrosis		^141^

**Table 4:** Examples of opposing functions of TG2 in cancer

**Cancer**	**Reports**	**Possible causation**	**References**
Progressive effect	Increased drug resistance and metastasis in breast cancer cells	Different cell types	^33, 142^
	Stabilization of adhesive interactions for metastasis in melanoma cells	Different cell types	^144^
	Anti-apoptotic effect in RA- or HPR-treated NIH3T3, HL-60, and embryonic lung fibroblast cells	Retention of Rb protein action	^150^
	Attenuated apoptosis in thapsigargin-treated HEK293 cells	Different chemopreventive agents and cell types	^151^
	TG2 enhances EGFR expression and transformation in glioblastomas	Interference with EGFR downregulation	^148^
Suppressive effect	Suppression of tumor progression and metastasis	Reduced metastatic activity via excess stabilization/accumulation of ECM crosslinking	^145, 146^
	Chemoprevention by retinoid	Different chemopreventive agents and cell types	^75^
	Growth arrest/apoptosis in RA-treated U937 cells	Lost activity of Rb protein	^149^
	Phosphorylation of Rb protein in MCF-7		^37^
No effect	Cell growth/apoptosis in TG2-transfected fibrosarcoma cell	Different cell types	^146^

**Table 5:** Examples of opposing functions of TG2 in the pancreas

**Pancreas**	**Reports**	**Possible causation**	**References**
Promotive effect	Glucose-stimulated insulin release	Different functional activity of TG2	^152, 153, 154, 158, 159^
	Insulin secretion induced by RA and Ca^2+^		^155, 156^
No effect	Insulin secretion induced by either cAMP or the phorbol ester PMAIntraperitoneal glucose tolerance tests and insulin tolerance tests	Different functional activity of TG2Different background of TG2 knockout miceBroad biological effects of TG inhibitors	^157, 160^

**Table 6:** Examples of opposing functions of TG2 in blood vessel formation

**Blood vessel**	**Reports**	**Possible causation**	**References**
Positive role	Formation and stability of blood vessels	Promotion of angiogenic process via VEGF signals in endothelium	^161, 162^
	Migration and tubule formation of HUVEC	TG2 inhibition results in reduced FN deposition, matrix-bound VEGFA, phosphorylation of VEGF receptor 2, and then suppresses migration and tubule formation	^30^
	Endothelial sprouting, migration of both endothelial and vascular mesenchymal cells, and organization of actin cytoskeleton	Promotion of the angiogenic process in endothelial and mesenchymal cells	^164^
	Cell spreading and adhesion in the human endothelial cell line ECV304	Adhesion activity with integrin or FN	^113, 166^
	Colocalization and binding with endostatin	Promotion of angiogenesis via endostatin-TG2 binding	^167, 168^
Negative role	Inhibition of angiogenesis and tumor growth via matrix changes through an intratumoral injection of TG2 into mice	Reduced organized vasculature via excess stabilization/accumulation of ECM crosslinking	^171^
	RA-mediated apoptosis in vascular smooth muscle cells	Effect in smooth muscle cells	^54^

## Abbreviations

TG: Transglutaminase
ECM: Extracellular matrix
ER: Endoplasmic reticulum
FN: Fibronectin
NF-*κ*B: Nuclear factor-kappa B
RA: Retinoic acid
RAR: Retinoic acid receptor
PDI: Protein disulfide isomerase
TGF-*β*: Transforming growth factor-*β*
ASH: Alcoholic steatohepatitis
NASH: Non-alcoholic steatohepatitis

## References

[bib1] Fesus L, Piacentini M. Transglutaminase 2: an enigmatic enzyme with diverse functions. Trends Biochem Sci 2002; 27: 534–539. 12368090 10.1016/s0968-0004(02)02182-5

[bib2] Iismaa SE, Mearns BM, Lorand L, Graham RM. Transglutaminases and disease: lessons from genetically engineered mouse models and inherited disorders. Physiol Rev 2009; 89: 991–1023. 19584319 10.1152/physrev.00044.2008

[bib3] Lorand L, Graham RM. Transglutaminases: crosslinking enzymes with pleiotropic functions. Nat Rev Mol Cell Biol 2003; 4: 140–156. 12563291 10.1038/nrm1014

[bib4] Iismaa SE, Chung L, Wu MJ, Teller DC, Yee VC, Graham RM. The core domain of the tissue transglutaminase Gh hydrolyzes GTP and ATP. Biochemistry 1997; 36: 11655–11664. 9305955 10.1021/bi970545e

[bib5] Lai TS, Slaughter TF, Koropchak CM, Haroon ZA, Greenberg CS. C-terminal deletion of human tissue transglutaminase enhances magnesium-dependent GTP/ATPase activity. J Biol Chem 1996; 271: 31191–31195. 8940119 10.1074/jbc.271.49.31191

[bib6] Hasegawa G, Suwa M, Ichikawa Y, Ohtsuka T, Kumagai S, Kikuchi M et al. A novel function of tissue-type transglutaminase: protein disulphide isomerase. Biochem J 2003; 373: 793–803. 12737632 10.1042/BJ20021084 PMC1223550

[bib7] Mishra S, Murphy LJ. Tissue transglutaminase has intrinsic kinase activity: identification of transglutaminase 2 as an insulin-like growth factor-binding protein-3 kinase. J Biol Chem 2004; 279: 23863–23868. 15069073 10.1074/jbc.M311919200

[bib8] Griffin M, Casadio R, Bergamini CM. Transglutaminases: nature's biological glues. Biochem J 2002; 368: 377–396. 12366374 10.1042/BJ20021234 PMC1223021

[bib9] Eckert RL, Kaartinen MT, Nurminskaya M, Belkin AM, Colak G, Johnson GV et al. Transglutaminase regulation of cell function. Physiol Rev 2014; 94: 383–417. 24692352 10.1152/physrev.00019.2013 PMC4044299

[bib10] Kanchan K, Fuxreiter M, Fesus L. Physiological, pathological, and structural implications of non-enzymatic protein-protein interactions of the multifunctional human transglutaminase 2. Cell Mol Life Sci 2015; 72: 3009–3035. 25943306 10.1007/s00018-015-1909-z PMC11113818

[bib11] Kuo TF, Tatsukawa H, Matsuura T, Nagatsuma K, Hirose S, Kojima S. Free fatty acids induce transglutaminase 2-dependent apoptosis in hepatocytes via ER stress-stimulated PERK pathways. J Cell Physiol 2012; 227: 1130–1137. 21567402 10.1002/jcp.22833

[bib12] Tatsukawa H, Fukaya Y, Frampton G, Martinez-Fuentes A, Suzuki K, Kuo TF et al. Role of transglutaminase 2 in liver injury via cross-linking and silencing of transcription factor Sp1. Gastroenterology 2009; 136: e1710. 10.1053/j.gastro.2009.01.007 PMC4960455 19208340

[bib13] Tatsukawa H, Kojima S. Recent advances in understanding the roles of transglutaminase 2 in alcoholic steatohepatitis. Cell Biol Int 2010; 34: 325–334. 20192918 10.1042/CBI20090130

[bib14] Hitomi K, Kojima S, Fesus L (eds). Transglutaminases, Multiple Functional Modifiers and Targets for New Drug Discovery, 1st edn. Springer Japan: Tokyo, Japan, 2015.

[bib15] Fesus L, Szondy Z. Transglutaminase 2 in the balance of cell death and survival. FEBS Lett 2005; 579: 3297–3302. 15943974 10.1016/j.febslet.2005.03.063

[bib16] Kiraly R, Demeny M, Fesus L. Protein transamidation by transglutaminase 2 in cells: a disputed Ca2+-dependent action of a multifunctional protein. FEBS J 2011; 278: 4717–4739. 21902809 10.1111/j.1742-4658.2011.08345.x

[bib17] Tee AE, Marshall GM, Liu PY, Xu N, Haber M, Norris MD et al. Opposing effects of two tissue transglutaminase protein isoforms in neuroblastoma cell differentiation. J Biol Chem 2010; 285: 3561–3567. 20007697 10.1074/jbc.M109.053041 PMC2823496

[bib18] Kiraly R, Barta E, Fesus L. Polymorphism of transglutaminase 2: unusually low frequency of genomic variants with deficient functions. Amino Acids 2013; 44: 215–225. 22160262 10.1007/s00726-011-1194-6

[bib19] Feng JF, Rhee SG, Im MJ. Evidence that phospholipase delta1 is the effector in the Gh (transglutaminase II)-mediated signaling. J Biol Chem 1996; 271: 16451–16454. 8663582 10.1074/jbc.271.28.16451

[bib20] Sarang Z, Molnar P, Nemeth T, Gomba S, Kardon T, Melino G et al. Tissue transglutaminase (TG2) acting as G protein protects hepatocytes against Fas-mediated cell death in mice. Hepatology 2005; 42: 578–587. 16108039 10.1002/hep.20812

[bib21] Huo J, Metz SA, Li G. Role of tissue transglutaminase in GTP depletion-induced apoptosis of insulin-secreting (HIT-T15) cells. Biochem Pharmacol 2003; 66: 213–223. 12826264 10.1016/s0006-2952(03)00262-4

[bib22] Monsonego A, Friedmann I, Shani Y, Eisenstein M, Schwartz M. GTP-dependent conformational changes associated with the functional switch between Galpha and cross-linking activities in brain-derived tissue transglutaminase. J Mol Biol 1998; 282: 713–720. 9743620 10.1006/jmbi.1998.2052

[bib23] Begg GE, Carrington L, Stokes PH, Matthews JM, Wouters MA, Husain A et al. Mechanism of allosteric regulation of transglutaminase 2 by GTP. Proc Natl Acad Sci USA 2006; 103: 19683–19688. 17179049 10.1073/pnas.0609283103 PMC1750866

[bib24] Aeschlimann D, Paulsson M, Mann K. Identification of Gln726 in nidogen as the amine acceptor in transglutaminase-catalyzed cross-linking of laminin-nidogen complexes. J Biol Chem 1992; 267: 11316–11321. 1350783

[bib25] Kaartinen MT, Pirhonen A, Linnala-Kankkunen A, Maenpaa PH. Transglutaminase-catalyzed cross-linking of osteopontin is inhibited by osteocalcin. J Biol Chem 1997; 272: 22736–22741. 9278432 10.1074/jbc.272.36.22736

[bib26] Kleman JP, Aeschlimann D, Paulsson M, van der Rest M. Transglutaminase-catalyzed cross-linking of fibrils of collagen V/XI in A204 rhabdomyosarcoma cells. Biochemistry 1995; 34: 13768–13775. 7577969 10.1021/bi00042a007

[bib27] Martinez J, Chalupowicz DG, Roush RK, Sheth A, Barsigian C. Transglutaminase-mediated processing of fibronectin by endothelial cell monolayers. Biochemistry 1994; 33: 2538–2545. 7906954 10.1021/bi00175a024

[bib28] Akimov SS, Belkin AM. Cell-surface transglutaminase promotes fibronectin assembly via interaction with the gelatin-binding domain of fibronectin: a role in TGFbeta-dependent matrix deposition. J Cell Sci 2001; 114: 2989–3000. 11686302 10.1242/jcs.114.16.2989

[bib29] Wang Z, Collighan RJ, Gross SR, Danen EH, Orend G, Telci D et al. RGD-independent cell adhesion via a tissue transglutaminase-fibronectin matrix promotes fibronectin fibril deposition and requires syndecan-4/2 alpha5beta1 integrin co-signaling. J Biol Chem 2010; 285: 40212–40229. 20929862 10.1074/jbc.M110.123703 PMC3001003

[bib30] Wang Z, Telci D, Griffin M. Importance of syndecan-4 and syndecan -2 in osteoblast cell adhesion and survival mediated by a tissue transglutaminase-fibronectin complex. Exp Cell Res 2011; 317: 367–381. 21036168 10.1016/j.yexcr.2010.10.015

[bib31] Song H, Chang W, Lim S, Seo HS, Shim CY, Park S et al. Tissue transglutaminase is essential for integrin-mediated survival of bone marrow-derived mesenchymal stem cells. Stem Cells 2007; 25: 1431–1438. 17347495 10.1634/stemcells.2006-0467

[bib32] Khanna M, Chelladurai B, Gavini A, Li L, Shao M, Courtney D et al. Targeting ovarian tumor cell adhesion mediated by tissue transglutaminase. Mol Cancer Ther 2011; 10: 626–636. 21330459 10.1158/1535-7163.MCT-10-0912

[bib33] Mehta K, Kumar A, Kim HI. Transglutaminase 2: a multi-tasking protein in the complex circuitry of inflammation and cancer. Biochem Pharmacol 2010; 80: 1921–1929. 20599779 10.1016/j.bcp.2010.06.029

[bib34] Malorni W, Farrace MG, Matarrese P, Tinari A, Ciarlo L, Mousavi-Shafaei P et al. The adenine nucleotide translocator 1 acts as a type 2 transglutaminase substrate: implications for mitochondrial-dependent apoptosis. Cell Death Differ 2009; 16: 1480–1492. 19644512 10.1038/cdd.2009.100

[bib35] Mishra S, Murphy LJ. The p53 oncoprotein is a substrate for tissue transglutaminase kinase activity. Biochem Biophys Res Commun 2006; 339: 726–730. 16313886 10.1016/j.bbrc.2005.11.071

[bib36] Mishra S, Saleh A, Espino PS, Davie JR, Murphy LJ. Phosphorylation of histones by tissue transglutaminase. J Biol Chem 2006; 281: 5532–5538. 16407273 10.1074/jbc.M506864200

[bib37] Mishra S, Melino G, Murphy LJ. Transglutaminase 2 kinase activity facilitates protein kinase A-induced phosphorylation of retinoblastoma protein. J Biol Chem 2007; 282: 18108–18115. 17478427 10.1074/jbc.M607413200

[bib38] Beninati S, Gentile V, Caraglia M, Lentini A, Tagliaferri P, Abbruzzese A. Tissue transglutaminase expression affects hypusine metabolism in BALB/c 3T3 cells. FEBS Lett 1998; 437: 34–38. 9804167 10.1016/s0014-5793(98)01191-0

[bib39] Beninati S, Nicolini L, Jakus J, Passeggio A, Abbruzzese A. Identification of a substrate site for transglutaminases on the human protein synthesis initiation factor 5 A. Biochem J 1995; 305: 725–728. 7848270 10.1042/bj3050725 PMC1136319

[bib40] Singh US, Li Q, Cerione R. Identification of the eukaryotic initiation factor 5A as a retinoic acid-stimulated cellular binding partner for tissue transglutaminase II. J Biol Chem 1998; 273: 1946–1950. 9442029 10.1074/jbc.273.4.1946

[bib41] Curro M, Condello S, Caccamo D, Ferlazzo N, Parisi G, Ientile R. Homocysteine-induced toxicity increases TG2 expression in Neuro2a cells. Amino Acids 2009; 36: 725–730. 18594946 10.1007/s00726-008-0122-x

[bib42] Nicholas B, Smethurst P, Verderio E, Jones R, Griffin M. Cross-linking of cellular proteins by tissue transglutaminase during necrotic cell death: a mechanism for maintaining tissue integrity. Biochem J 2003; 371: 413–422. 12533191 10.1042/BJ20021949 PMC1223304

[bib43] Kim SY. Transglutaminase 2 in inflammation. Front Biosci 2006; 11: 3026–3035. 16720373 10.2741/2030

[bib44] Amendola A, Rodolfo C, Di Caro A, Ciccosanti F, Falasca L, Piacentini M. "Tissue" transglutaminase expression in HIV-infected cells: an enzyme with an antiviral effect? Ann NY Acad Sci 2001; 946: 108–120. 11762979

[bib45] Rodolfo C, Mormone E, Matarrese P, Ciccosanti F, Farrace MG, Garofano E et al. Tissue transglutaminase is a multifunctional BH3-only protein. J Biol Chem 2004; 279: 54783–54792. 15485857 10.1074/jbc.M410938200

[bib46] Szegezdi E, Szondy Z, Nagy L, Nemes Z, Friis RR, Davies PJ et al. Apoptosis-linked *in vivo* regulation of the tissue transglutaminase gene promoter. Cell Death Differ 2000; 7: 1225–1233. 11175260 10.1038/sj.cdd.4400751

[bib47] Antonyak MA, Miller AM, Jansen JM, Boehm JE, Balkman CE, Wakshlag JJ et al. Augmentation of tissue transglutaminase expression and activation by epidermal growth factor inhibit doxorubicin-induced apoptosis in human breast cancer cells. J Biol Chem 2004; 279: 41461–41467. 15272014 10.1074/jbc.M404976200

[bib48] Herman JF, Mangala LS, Mehta K. Implications of increased tissue transglutaminase (TG2) expression in drug-resistant breast cancer (MCF-7) cells. Oncogene 2006; 25: 3049–3058. 16449978 10.1038/sj.onc.1209324

[bib49] Park KS, Kim DS, Jeong KC, Kim SY. Increase in transglutaminase 2 expression is associated with NF-kappaB activation in breast cancer tissues. Front Biosci (Landmark Ed) 2009; 14: 1945–1951. 19273176 10.2741/3354

[bib50] Ritter SJ, Davies PJ. Identification of a transforming growth factor-beta1/bone morphogenetic protein 4 (TGF-beta1/BMP4) response element within the mouse tissue transglutaminase gene promoter. J Biol Chem 1998; 273: 12798–12806. 9582307 10.1074/jbc.273.21.12798

[bib51] Johnson K, Hashimoto S, Lotz M, Pritzker K, Terkeltaub R. Interleukin-1 induces pro-mineralizing activity of cartilage tissue transglutaminase and factor XIIIa. Am J Pathol 2001; 159: 149–163. 11438463 10.1016/S0002-9440(10)61682-3 PMC1850418

[bib52] Suto N, Ikura K, Sasaki R. Expression induced by interleukin-6 of tissue-type transglutaminase in human hepatoblastoma HepG2 cells. J Biol Chem 1993; 268: 7469–7473. 8096510

[bib53] Kuncio GS, Tsyganskaya M, Zhu J, Liu SL, Nagy L, Thomazy V et al. TNF-alpha modulates expression of the tissue transglutaminase gene in liver cells. Am J Physiol 1998; 274: G240–G245. 9486175 10.1152/ajpgi.1998.274.2.G240

[bib54] Ou H, Haendeler J, Aebly MR, Kelly LA, Cholewa BC, Koike G et al. Retinoic acid-induced tissue transglutaminase and apoptosis in vascular smooth muscle cells. Circ Res 2000; 87: 881–887. 11073883 10.1161/01.res.87.10.881

[bib55] Shimada J, Suzuki Y, Kim SJ, Wang PC, Matsumura M, Kojima S. Transactivation via RAR/RXR-Sp1 interaction: characterization of binding between Sp1 and GC box motif. Mol Endocrinol 2001; 15: 1677–1692. 11579201 10.1210/mend.15.10.0707

[bib56] Balajthy Z, Csomos K, Vamosi G, Szanto A, Lanotte M, Fesus L. Tissue-transglutaminase contributes to neutrophil granulocyte differentiation and functions. Blood 2006; 108: 2045–2054. 16763214 10.1182/blood-2004-02-007948

[bib57] Kojima S, Nara K, Rifkin DB. Requirement for transglutaminase in the activation of latent transforming growth factor-beta in bovine endothelial cells. J Cell Biol 1993; 121: 439–448. 8096847 10.1083/jcb.121.2.439 PMC2200108

[bib58] Nunes I, Gleizes PE, Metz CN, Rifkin DB. Latent transforming growth factor-beta binding protein domains involved in activation and transglutaminase-dependent cross-linking of latent transforming growth factor-beta. J Cell Biol 1997; 136: 1151–1163. 9060478 10.1083/jcb.136.5.1151 PMC2132473

[bib59] Oh K, Park HB, Byoun OJ, Shin DM, Jeong EM, Kim YW et al. Epithelial transglutaminase 2 is needed for T cell interleukin-17 production and subsequent pulmonary inflammation and fibrosis in bleomycin-treated mice. J Exp Med 2011; 208: 1707–1719. 21746810 10.1084/jem.20101457 PMC3149214

[bib60] Rosenthal AK, Gohr CM, Henry LA, Le M. Participation of transglutaminase in the activation of latent transforming growth factor beta1 in aging articular cartilage. Arthritis Rheum 2000; 43: 1729–1733. 10943862 10.1002/1529-0131(200008)43:8<1729::AID-ANR8>3.0.CO;2-0

[bib61] Shweke N, Boulos N, Jouanneau C, Vandermeersch S, Melino G, Dussaule JC et al. Tissue transglutaminase contributes to interstitial renal fibrosis by favoring accumulation of fibrillar collagen through TGF-beta activation and cell infiltration. Am J Pathol 2008; 173: 631–642. 18688035 10.2353/ajpath.2008.080025 PMC2527082

[bib62] Szondy Z, Sarang Z, Molnar P, Nemeth T, Piacentini M, Mastroberardino PG et al. Transglutaminase 2^−/−^ mice reveal a phagocytosis-associated crosstalk between macrophages and apoptotic cells. Proc Natl Acad Sci USA 2003; 100: 7812–7817. 12810961 10.1073/pnas.0832466100 PMC164670

[bib63] Telci D, Collighan RJ, Basaga H, Griffin M. Increased TG2 expression can result in induction of transforming growth factor beta1, causing increased synthesis and deposition of matrix proteins, which can be regulated by nitric oxide. J Biol Chem 2009; 284: 29547–29558. 19657147 10.1074/jbc.M109.041806 PMC2785588

[bib64] Chaturvedi MM, Sung B, Yadav VR, Kannappan R, Aggarwal BB. NF-kappaB addiction and its role in cancer: 'one size does not fit all'. Oncogene 2011; 30: 1615–1630. 21170083 10.1038/onc.2010.566 PMC3141287

[bib65] Kim JM, Voll RE, Ko C, Kim DS, Park KS, Kim SY. A new regulatory mechanism of NF-kappaB activation by I-kappaBbeta in cancer cells. J Mol Biol 2008; 384: 756–765. 18950638 10.1016/j.jmb.2008.10.010

[bib66] Park SS, Kim JM, Kim DS, Kim IH, Kim SY. Transglutaminase 2 mediates polymer formation of I-kappaBalpha through C-terminal glutamine cluster. J Biol Chem 2006; 281: 34965–34972. 16987813 10.1074/jbc.M604150200

[bib67] Lai TS, Hausladen A, Slaughter TF, Eu JP, Stamler JS, Greenberg CS. Calcium regulates S-nitrosylation, denitrosylation, and activity of tissue transglutaminase. Biochemistry 2001; 40: 4904–4910. 11305905 10.1021/bi002321t

[bib68] Santhanam L, Tuday EC, Webb AK, Dowzicky P, Kim JH, Oh YJ et al. Decreased S-nitrosylation of tissue transglutaminase contributes to age-related increases in vascular stiffness. Circ Res 2010; 107: 117–125. 20489165 10.1161/CIRCRESAHA.109.215228

[bib69] Luciani A, Villella VR, Vasaturo A, Giardino I, Raia V, Pettoello-Mantovani M et al. SUMOylation of tissue transglutaminase as link between oxidative stress and inflammation. J Immunol 2009; 183: 2775–2784. 19625650 10.4049/jimmunol.0900993

[bib70] Amendola A, Gougeon ML, Poccia F, Bondurand A, Fesus L, Piacentini M. Induction of "tissue" transglutaminase in HIV pathogenesis: evidence for high rate of apoptosis of CD4+ T lymphocytes and accessory cells in lymphoid tissues. Proc Natl Acad Sci USA 1996; 93: 11057–11062. 8855308 10.1073/pnas.93.20.11057 PMC38283

[bib71] Fesus L. Biochemical events in naturally occurring forms of cell death. FEBS Lett 1993; 328: 1–5. 8344412 10.1016/0014-5793(93)80952-q

[bib72] Melino G, Annicchiarico-Petruzzelli M, Piredda L, Candi E, Gentile V, Davies PJ et al. Tissue transglutaminase and apoptosis: sense and antisense transfection studies with human neuroblastoma cells. Mol Cell Biol 1994; 14: 6584–6596. 7935379 10.1128/mcb.14.10.6584-6596.1994 PMC359188

[bib73] Oliverio S, Amendola A, Rodolfo C, Spinedi A, Piacentini M. Inhibition of "tissue" transglutaminase increases cell survival by preventing apoptosis. J Biol Chem 1999; 274: 34123–34128. 10567382 10.1074/jbc.274.48.34123

[bib74] Tucholski J, Johnson GV. Tissue transglutaminase differentially modulates apoptosis in a stimuli-dependent manner. J Neurochem 2002; 81: 780–791. 12065637 10.1046/j.1471-4159.2002.00859.x

[bib75] Tatsukawa H, Sano T, Fukaya Y, Ishibashi N, Watanabe M, Okuno M et al. Dual induction of caspase 3- and transglutaminase-dependent apoptosis by acyclic retinoid in hepatocellular carcinoma cells. Mol Cancer 2011; 10: 4. 21214951 10.1186/1476-4598-10-4 PMC3024303

[bib76] Kweon SM, Lee ZW, Yi SJ, Kim YM, Han JA, Paik SG et al. Protective role of tissue transglutaminase in the cell death induced by TNF-alpha in SH-SY5Y neuroblastoma cells. J Biochem Mol Biol 2004; 37: 185–191. 15469694 10.5483/bmbrep.2004.37.2.185

[bib77] Antonyak MA, Singh US, Lee DA, Boehm JE, Combs C, Zgola MM et al. Effects of tissue transglutaminase on retinoic acid-induced cellular differentiation and protection against apoptosis. J Biol Chem 2001; 276: 33582–33587. 11438548 10.1074/jbc.M105318200

[bib78] Gundemir S, Johnson GV. Intracellular localization and conformational state of transglutaminase 2: implications for cell death. PLoS One 2009; 4: e6123. 19568436 10.1371/journal.pone.0006123 PMC2701606

[bib79] Hwang IK, Yoo KY, Yi SS, Kim IY, Hwang HS, Lee KY et al. Expression of tissue-type transglutaminase (tTG) and the effect of tTG inhibitor on the hippocampal CA1 region after transient ischemia in gerbils. Brain Res 2009; 1263: 134–142. 19368835 10.1016/j.brainres.2009.01.038

[bib80] Ientile R, Caccamo D, Marciano MC, Curro M, Mannucci C, Campisi A et al. Transglutaminase activity and transglutaminase mRNA transcripts in gerbil brain ischemia. Neurosci Lett 2004; 363: 173–177. 15172109 10.1016/j.neulet.2004.04.003

[bib81] Takano K, Shiraiwa K, Moriyama M, Nakamura Y. Transglutaminase 2 expression induced by lipopolysaccharide stimulation together with NO synthase induction in cultured astrocytes. Neurochem Int 2010; 57: 812–818. 20817067 10.1016/j.neuint.2010.08.019

[bib82] Saiki R, Park H, Ishii I, Yoshida M, Nishimura K, Toida T et al. Brain infarction correlates more closely with acrolein than with reactive oxygen species. Biochem Biophys Res Commun 2011; 404: 1044–1049. 21187074 10.1016/j.bbrc.2010.12.107

[bib83] Filiano AJ, Bailey CD, Tucholski J, Gundemir S, Johnson GV. Transglutaminase 2 protects against ischemic insult, interacts with HIF1beta, and attenuates HIF1 signaling. FASEB J 2008; 22: 2662–2675. 18375543 10.1096/fj.07-097709 PMC2493449

[bib84] Filiano AJ, Tucholski J, Dolan PJ, Colak G, Johnson GV. Transglutaminase 2 protects against ischemic stroke. Neurobiol Dis 2010; 39: 334–343. 20451610 10.1016/j.nbd.2010.04.018 PMC2917584

[bib85] Boyd JM, Malstrom S, Subramanian T, Venkatesh LK, Schaeper U, Elangovan B et al. Adenovirus E1B 19 kDa and Bcl-2 proteins interact with a common set of cellular proteins. Cell 1994; 79: 341–351. 7954800 10.1016/0092-8674(94)90202-x

[bib86] Oda E, Ohki R, Murasawa H, Nemoto J, Shibue T, Yamashita T et al. Noxa, a BH3-only member of the Bcl-2 family and candidate mediator of p53-induced apoptosis. Science 2000; 288: 1053–1058. 10807576 10.1126/science.288.5468.1053

[bib87] Jeitner TM, Pinto JT, Krasnikov BF, Horswill M, Cooper AJ. Transglutaminases and neurodegeneration. J Neurochem 2009; 109(Suppl 1):160–166. 19393023 10.1111/j.1471-4159.2009.05843.x PMC2752967

[bib88] Dudek SM, Johnson GV. Transglutaminase facilitates the formation of polymers of the beta-amyloid peptide. Brain Res 1994; 651: 129–133. 7922559 10.1016/0006-8993(94)90688-2

[bib89] Miller ML, Johnson GV. Transglutaminase cross-linking of the tau protein. J Neurochem 1995; 65: 1760–1770. 7561874 10.1046/j.1471-4159.1995.65041760.x

[bib90] Tucholski J, Kuret J, Johnson GV. Tau is modified by tissue transglutaminase *in situ*: possible functional and metabolic effects of polyamination. J Neurochem 1999; 73: 1871–1880. 10537045

[bib91] Antonyak MA, Jansen JM, Miller AM, Ly TK, Endo M, Cerione RA. Two isoforms of tissue transglutaminase mediate opposing cellular fates. Proc Natl Acad Sci USA 2006; 103: 18609–18614. 17116873 10.1073/pnas.0604844103 PMC1654133

[bib92] Citron BA, SantaCruz KS, Davies PJ, Festoff BW. Intron-exon swapping of transglutaminase mRNA and neuronal Tau aggregation in Alzheimer's disease. J Biol Chem 2001; 276: 3295–3301. 11013236 10.1074/jbc.M004776200

[bib93] Wolf J, Jager C, Lachmann I, Schonknecht P, Morawski M, Arendt T et al. Tissue transglutaminase is not a biochemical marker for Alzheimer's disease. Neurobiol Aging 2013; 34: 2495–2498. 23747046 10.1016/j.neurobiolaging.2013.05.008

[bib94] Zainelli GM, Ross CA, Troncoso JC, Muma NA. Transglutaminase cross-links in intranuclear inclusions in Huntington disease. J Neuropathol Exp Neurol 2003; 62: 14–24. 12528814 10.1093/jnen/62.1.14

[bib95] Igarashi S, Koide R, Shimohata T, Yamada M, Hayashi Y, Takano H et al. Suppression of aggregate formation and apoptosis by transglutaminase inhibitors in cells expressing truncated DRPLA protein with an expanded polyglutamine stretch. Nat Genet 1998; 18: 111–117. 9462738 10.1038/ng0298-111

[bib96] Bailey CD, Johnson GV. Tissue transglutaminase contributes to disease progression in the R6/2 Huntington's disease mouse model via aggregate-independent mechanisms. J Neurochem 2005; 92: 83–92. 15606898 10.1111/j.1471-4159.2004.02839.x

[bib97] Mastroberardino PG, Iannicola C, Nardacci R, Bernassola F, De Laurenzi V, Melino G et al. 'Tissue' transglutaminase ablation reduces neuronal death and prolongs survival in a mouse model of Huntington's disease. Cell Death Differ 2002; 9: 873–880. 12181738 10.1038/sj.cdd.4401093

[bib98] D'Eletto M, Farrace MG, Falasca L, Reali V, Oliverio S, Melino G et al. Transglutaminase 2 is involved in autophagosome maturation. Autophagy 2009; 5: 1145–1154. 19955852 10.4161/auto.5.8.10040

[bib99] Lesort M, Lee M, Tucholski J, Johnson GV. Cystamine inhibits caspase activity. Implications for the treatment of polyglutamine disorders. J Biol Chem 2003; 278: 3825–3830. 12458211 10.1074/jbc.M205812200

[bib100] Mastroberardino PG, Piacentini M. Type 2 transglutaminase in Huntington's disease: a double-edged sword with clinical potential. J Intern Med 2010; 268: 419–431. 20964734 10.1111/j.1365-2796.2010.02275.x PMC3073231

[bib101] McConoughey SJ, Basso M, Niatsetskaya ZV, Sleiman SF, Smirnova NA, Langley BC et al. Inhibition of transglutaminase 2 mitigates transcriptional dysregulation in models of Huntington disease. EMBO Mol Med 2010; 2: 349–370. 20665636 10.1002/emmm.201000084 PMC3068019

[bib102] Colby DW, Cassady JP, Lin GC, Ingram VM, Wittrup KD. Stochastic kinetics of intracellular huntingtin aggregate formation. Nat Chem Biol 2006; 2: 319–323. 16699519 10.1038/nchembio792

[bib103] Kazemi-Esfarjani P, La Spada AR. Deja vu with a twist: transglutaminases in bioenergetics and transcriptional dysfunction in Huntington's disease. EMBO Mol Med 2010; 2: 335–337. 20730854 10.1002/emmm.201000092 PMC3377338

[bib104] Chen-Plotkin AS, Sadri-Vakili G, Yohrling GJ, Braveman MW, Benn CL, Glajch KE et al. Decreased association of the transcription factor Sp1 with genes downregulated in Huntington's disease. Neurobiol Dis 2006; 22: 233–241. 16442295 10.1016/j.nbd.2005.11.001

[bib105] Dunah AW, Jeong H, Griffin A, Kim YM, Standaert DG, Hersch SM et al. Sp1 and TAFII130 transcriptional activity disrupted in early Huntington's disease. Science 2002; 296: 2238–2243. 11988536 10.1126/science.1072613

[bib106] Li SH, Cheng AL, Zhou H, Lam S, Rao M, Li H et al. Interaction of Huntington disease protein with transcriptional activator Sp1. Mol Cell Biol 2002; 22: 1277–1287. 11839795 10.1128/mcb.22.5.1277-1287.2002 PMC134707

[bib107] Zuccato C, Ciammola A, Rigamonti D, Leavitt BR, Goffredo D, Conti L et al. Loss of huntingtin-mediated BDNF gene transcription in Huntington's disease. Science 2001; 293: 493–498. 11408619 10.1126/science.1059581

[bib108] Connor MK, Irrcher I, Hood DA. Contractile activity-induced transcriptional activation of cytochrome C involves Sp1 and is proportional to mitochondrial ATP synthesis in C2C12 muscle cells. J Biol Chem 2001; 276: 15898–15904. 11279044 10.1074/jbc.M100272200

[bib109] Evans MJ, Scarpulla RC. Interaction of nuclear factors with multiple sites in the somatic cytochrome c promoter. Characterization of upstream NRF-1, ATF, and intron Sp1 recognition sequences. J Biol Chem 1989; 264: 14361–14368. 2547796

[bib110] Munsie L, Caron N, Atwal RS, Marsden I, Wild EJ, Bamburg JR et al. Mutant huntingtin causes defective actin remodeling during stress: defining a new role for transglutaminase 2 in neurodegenerative disease. Hum Mol Genet 2011; 20: 1937–1951. 21355047 10.1093/hmg/ddr075 PMC3080606

[bib111] McGough A, Pope B, Chiu W, Weeds A. Cofilin changes the twist of F-actin: implications for actin filament dynamics and cellular function. J Cell Biol 1997; 138: 771–781. 9265645 10.1083/jcb.138.4.771 PMC2138052

[bib112] Belkin AM. Extracellular TG2: emerging functions and regulation. FEBS J 2011; 278: 4704–4716. 21902810 10.1111/j.1742-4658.2011.08346.x PMC3228878

[bib113] Gaudry CA, Verderio E, Jones RA, Smith C, Griffin M. Tissue transglutaminase is an important player at the surface of human endothelial cells: evidence for its externalization and its colocalization with the beta(1) integrin. Exp Cell Res 1999; 252: 104–113. 10502403 10.1006/excr.1999.4633

[bib114] Andringa G, Lam KY, Chegary M, Wang X, Chase TN, Bennett MC. Tissue transglutaminase catalyzes the formation of alpha-synuclein crosslinks in Parkinson's disease. FASEB J 2004; 18: 932–934. 15001552 10.1096/fj.03-0829fje

[bib115] Junn E, Ronchetti RD, Quezado MM, Kim SY, Mouradian MM. Tissue transglutaminase-induced aggregation of alpha-synuclein: Implications for Lewy body formation in Parkinson's disease and dementia with Lewy bodies. Proc Natl Acad Sci USA 2003; 100: 2047–2052. 12576551 10.1073/pnas.0438021100 PMC149956

[bib116] Chen CS, Wu CH, Lai YC, Lee WS, Chen HM, Chen RJ et al. NF-kappaB-activated tissue transglutaminase is involved in ethanol-induced hepatic injury and the possible role of propolis in preventing fibrogenesis. Toxicology 2008; 246: 148–157. 18295389 10.1016/j.tox.2008.01.009

[bib117] Grenard P, Bresson-Hadni S, El Alaoui S, Chevallier M, Vuitton DA, Ricard-Blum S. Transglutaminase-mediated cross-linking is involved in the stabilization of extracellular matrix in human liver fibrosis. J Hepatol 2001; 35: 367–375. 11592598 10.1016/s0168-8278(01)00135-0

[bib118] Mirza A, Liu SL, Frizell E, Zhu J, Maddukuri S, Martinez J et al. A role for tissue transglutaminase in hepatic injury and fibrogenesis, and its regulation by NF-kappaB. Am J Physiol 1997; 272: G281–G288. 9124352 10.1152/ajpgi.1997.272.2.G281

[bib119] Strnad P, Harada M, Siegel M, Terkeltaub RA, Graham RM, Khosla C et al. Transglutaminase 2 regulates mallory body inclusion formation and injury-associated liver enlargement. Gastroenterology 2007; 132: 1515–1526. 17408647 10.1053/j.gastro.2007.02.020

[bib120] Wu J, Liu SL, Zhu JL, Norton PA, Nojiri S, Hoek JB et al. Roles of tissue transglutaminase in ethanol-induced inhibition of hepatocyte proliferation and alpha 1-adrenergic signal transduction. J Biol Chem 2000; 275: 22213–22219. 10801782 10.1074/jbc.M000091200

[bib121] D'Argenio G, Amoruso DC, Mazzone G, Vitaglione P, Romano A, Ribecco MT et al. Garlic extract prevents CCl(4)-induced liver fibrosis in rats: The role of tissue transglutaminase. Dig Liver Dis 2010; 42: 571–577. 20004152 10.1016/j.dld.2009.11.002

[bib122] Courey AJ, Holtzman DA, Jackson SP, Tjian R. Synergistic activation by the glutamine-rich domains of human transcription factor Sp1. Cell 1989; 59: 827–836. 2512012 10.1016/0092-8674(89)90606-5

[bib123] Han JA, Park SC. Transglutaminase-dependent modulation of transcription factor Sp1 activity. Mol Cells 2000; 10: 612–618. 11211864 10.1007/s10059-000-0612-5

[bib124] Pascal E, Tjian R. Different activation domains of Sp1 govern formation of multimers and mediate transcriptional synergism. Genes Dev 1991; 5: 1646–1656. 1885006 10.1101/gad.5.9.1646

[bib125] Horino K, Nishiura H, Ohsako T, Shibuya Y, Hiraoka T, Kitamura N et al. A monocyte chemotactic factor, S19 ribosomal protein dimer, in phagocytic clearance of apoptotic cells. Lab Invest 1998; 78: 603–617. 9605185

[bib126] Toth B, Garabuczi E, Sarang Z, Vereb G, Vamosi G, Aeschlimann D et al. Transglutaminase 2 is needed for the formation of an efficient phagocyte portal in macrophages engulfing apoptotic cells. J Immunol 2009; 182: 2084–2092. 19201861 10.4049/jimmunol.0803444

[bib127] Suh GY, Ham HS, Lee SH, Choi JC, Koh WJ, Kim SY et al. A Peptide with anti-transglutaminase activity decreases lipopolysaccharide-induced lung inflammation in mice. Exp Lung Res 2006; 32: 43–53. 16809220 10.1080/01902140600691514

[bib128] Nardacci R, Lo Iacono O, Ciccosanti F, Falasca L, Addesso M, Amendola A et al. Transglutaminase type II plays a protective role in hepatic injury. Am J Pathol 2003; 162: 1293–1303. 12651621 10.1016/S0002-9440(10)63925-9 PMC1851212

[bib129] Wu J, Zern MA. Tissue transglutaminase, a key enzyme involved in liver diseases. Hepatol Res 2004; 29: 1–8. 15135339 10.1016/j.hepres.2004.02.007

[bib130] Friedman SL. Mechanisms of hepatic fibrogenesis. Gastroenterology 2008; 134: 1655–1669. 18471545 10.1053/j.gastro.2008.03.003 PMC2888539

[bib131] Gressner AM, Weiskirchen R, Breitkopf K, Dooley S. Roles of TGF-beta in hepatic fibrosis. Front Biosci 2002; 7: d793–d807. 11897555 10.2741/A812

[bib132] Le M, Gohr CM, Rosenthal AK. Transglutaminase participates in the incorporation of latent TGFbeta into the extracellular matrix of aging articular chondrocytes. Connect Tissue Res 2001; 42: 245–253. 11913769 10.3109/03008200109016839

[bib133] Hou XJ, Jin ZD, Jiang F, Zhu JW, Li ZS. Expression of Smad7 and Smad ubiquitin regulatory factor 2 in a rat model of chronic pancreatitis. J Dig Dis 2015; 16: 408–415. 25943897 10.1111/1751-2980.12253

[bib134] Borowiak M, Garratt AN, Wustefeld T, Strehle M, Trautwein C, Birchmeier C. Met provides essential signals for liver regeneration. Proc Natl Acad Sci USA 2004; 101: 10608–10613. 15249655 10.1073/pnas.0403412101 PMC490025

[bib135] Huh CG, Factor VM, Sanchez A, Uchida K, Conner EA, Thorgeirsson SS. Hepatocyte growth factor/c-met signaling pathway is required for efficient liver regeneration and repair. Proc Natl Acad Sci USA 2004; 101: 4477–4482. 15070743 10.1073/pnas.0306068101 PMC384772

[bib136] Giebeler A, Boekschoten MV, Klein C, Borowiak M, Birchmeier C, Gassler N et al. c-Met confers protection against chronic liver tissue damage and fibrosis progression after bile duct ligation in mice. Gastroenterology 2009; 137: 308 e291–e294. 10.1053/j.gastro.2009.01.068 19208365

[bib137] Kojima S, Kuo TF, Tatsukawa H, Hirose S. Induction of cross-linking and silencing of Sp1 by transglutaminase during liver injury in ASH and NASH via different ER stress pathways. Dig Dis 2010; 28: 715–721. 21525755 10.1159/000324278

[bib138] Diehl AM, Abdo S, Brown N. Supplemental putrescine reverses ethanol-associated inhibition of liver regeneration. Hepatology 1990; 12: 633–637. 2210666 10.1002/hep.1840120402

[bib139] Hsu TC, Huang CY, Chiang SY, Lai WX, Tsai CH, Tzang BS. Transglutaminase inhibitor cystamine alleviates the abnormality in liver from NZB/W F1 mice. Eur J Pharmacol 2008; 579: 382–389. 18031733 10.1016/j.ejphar.2007.10.059

[bib140] Shibley IAJr., Gavigan MD, Pennington SN. Ethanol's effect on tissue polyamines and ornithine decarboxylase activity: a concise review. Alcohol Clin Exp Res 1995; 19: 209–215. 7771652 10.1111/j.1530-0277.1995.tb01494.x

[bib141] Popov Y, Sverdlov DY, Sharma AK, Bhaskar KR, Li S, Freitag TL et al. Tissue transglutaminase does not affect fibrotic matrix stability or regression of liver fibrosis in mice. Gastroenterology 2011; 140: 1642–1652. 21277850 10.1053/j.gastro.2011.01.040 PMC3374132

[bib142] Mangala LS, Fok JY, Zorrilla-Calancha IR, Verma A, Mehta K. Tissue transglutaminase expression promotes cell attachment, invasion and survival in breast cancer cells. Oncogene 2007; 26: 2459–2470. 17043648 10.1038/sj.onc.1210035

[bib143] Iacobuzio-Donahue CA, Ashfaq R, Maitra A, Adsay NV, Shen-Ong GL, Berg K et al. Highly expressed genes in pancreatic ductal adenocarcinomas: a comprehensive characterization and comparison of the transcription profiles obtained from three major technologies. Cancer Res 2003; 63: 8614–8622. 14695172

[bib144] Kong L, Korthuis RJ. Melanoma cell adhesion to injured arterioles: mechanisms of stabilized tethering. Clin Exp Metastasis 1997; 15: 426–431. 9219731 10.1023/a:1018406422727

[bib145] Xu L, Hynes RO. GPR56 and TG2: possible roles in suppression of tumor growth by the microenvironment. Cell Cycle 2007; 6: 160–165. 17314516 10.4161/cc.6.2.3760

[bib146] Johnson TS, Knight CR, el-Alaoui S, Mian S, Rees RC, Gentile V et al. Transfection of tissue transglutaminase into a highly malignant hamster fibrosarcoma leads to a reduced incidence of primary tumour growth. Oncogene 1994; 9: 2935–2942. 7916148

[bib147] Shrestha RT, Shrestha H, Ishibashi R, Matsuura N, Kagechika T, Kose H et al. Molecular mechanism by which acyclic retinoid induces nuclear localization of transglutaminase 2 in human hepatocellular carcinoma cells. Cell Death Dis 2015; 6: e2002. 26633708 10.1038/cddis.2015.339 PMC4720877

[bib148] Zhang J, Antonyak MA, Singh G, Cerione RA. A mechanism for the upregulation of EGF receptor levels in glioblastomas. Cell Rep 2013; 3: 2008–2020. 23770238 10.1016/j.celrep.2013.05.021 PMC3742030

[bib149] Oliverio S, Amendola A, Di Sano F, Farrace MG, Fesus L, Nemes Z et al. Tissue transglutaminase-dependent posttranslational modification of the retinoblastoma gene product in promonocytic cells undergoing apoptosis. Mol Cell Biol 1997; 17: 6040–6048. 9315663 10.1128/mcb.17.10.6040 PMC232453

[bib150] Boehm JE, Singh U, Combs C, Antonyak MA, Cerione RA. Tissue transglutaminase protects against apoptosis by modifying the tumor suppressor protein p110 Rb. J Biol Chem 2002; 277: 20127–20130. 11956182 10.1074/jbc.C200147200

[bib151] Milakovic T, Tucholski J, McCoy E, Johnson GV. Intracellular localization and activity state of tissue transglutaminase differentially impacts cell death. J Biol Chem 2004; 279: 8715–8722. 14670969 10.1074/jbc.M308479200

[bib152] Bungay PJ, Owen RA, Coutts IC, Griffin M. A role for transglutaminase in glucose-stimulated insulin release from the pancreatic beta-cell. Biochem J 1986; 235: 269–278. 2874792 10.1042/bj2350269 PMC1146677

[bib153] Gomis R, Sener A, Malaisse-Lagae F, Malaisse WJ. Transglutaminase activity in pancreatic islets. Biochim Biophys Acta 1983; 760: 384–388. 6138101 10.1016/0304-4165(83)90378-1

[bib154] Sener A, Dunlop ME, Gomis R, Mathias PC, Malaisse-Lagae F, Malaisse WJ. Role of transglutaminase in insulin release. Study with glycine and sarcosine methylesters. Endocrinology 1985; 117: 237–242. 2408879 10.1210/endo-117-1-237

[bib155] Driscoll HK, Adkins CD, Chertow TE, Cordle MB, Matthews KA, Chertow BS. Vitamin A stimulation of insulin secretion: effects on transglutaminase mRNA and activity using rat islets and insulin-secreting cells. Pancreas 1997; 15: 69–77. 9211495

[bib156] Shibuya H, Sakai K, Kabir-Salmani M, Wachi Y, Iwashita M. Polymerization of insulin-like growth factor-binding protein-1 (IGFBP-1) potentiates IGF-I actions in placenta. J Cell Physiol 2011; 226: 434–439. 20672288 10.1002/jcp.22349

[bib157] Lindsay MA, Bungay PJ, Griffin M. Transglutaminase involvement in the secretion of insulin from electropermeabilised rat islets of Langerhans. Biosci Rep 1990; 10: 557–561. 1707691 10.1007/BF01116616

[bib158] Bernassola F, Federici M, Corazzari M, Terrinoni A, Hribal ML, De Laurenzi V et al. Role of transglutaminase 2 in glucose tolerance: knockout mice studies and a putative mutation in a MODY patient. FASEB J 2002; 16: 1371–1378. 12205028 10.1096/fj.01-0689com

[bib159] Porzio O, Massa O, Cunsolo V, Colombo C, Malaponti M, Bertuzzi F et al. Missense mutations in the TGM2 gene encoding transglutaminase 2 are found in patients with early-onset type 2 diabetes. Mutation in brief no. 982. Online. Hum Mutat 2007; 28: 1150. 10.1002/humu.9511 17939176

[bib160] Iismaa SE, Aplin M, Holman S, Yiu TW, Jackson K, Burchfield JG et al. Glucose homeostasis in mice is transglutaminase 2 independent. PLoS One 2013; 8: e63346. 23717413 10.1371/journal.pone.0063346 PMC3661676

[bib161] Dardik R, Inbal A. Complex formation between tissue transglutaminase II (tTG) and vascular endothelial growth factor receptor 2 (VEGFR-2): proposed mechanism for modulation of endothelial cell response to VEGF. Exp Cell Res 2006; 312: 2973–2982. 16914140 10.1016/j.yexcr.2006.05.019

[bib162] Haroon ZA, Hettasch JM, Lai TS, Dewhirst MW, Greenberg CS. Tissue transglutaminase is expressed, active, and directly involved in rat dermal wound healing and angiogenesis. FASEB J 1999; 13: 1787–1795. 10506581 10.1096/fasebj.13.13.1787

[bib163] Di Simone N, De Spirito M, Di Nicuolo F, Tersigni C, Castellani R, Silano M et al. Potential new mechanisms of placental damage in celiac disease: anti-transglutaminase antibodies impair human endometrial angiogenesis. Biol Reprod 2013; 89: 88. 23966323 10.1095/biolreprod.113.109637

[bib164] Myrsky E, Kaukinen K, Syrjanen M, Korponay-Szabo IR, Maki M, Lindfors K. Coeliac disease-specific autoantibodies targeted against transglutaminase 2 disturb angiogenesis. Clin Exp Immunol 2008; 152: 111–119. 18279443 10.1111/j.1365-2249.2008.03600.x PMC2384074

[bib165] Nadalutti CA, Korponay-Szabo IR, Kaukinen K, Griffin M, Maki M, Lindfors K. Celiac disease patient IgA antibodies induce endothelial adhesion and cell polarization defects via extracellular transglutaminase 2. Cell Mol Life Sci 2014; 71: 1315–1326. 23982754 10.1007/s00018-013-1455-5 PMC11113300

[bib166] Jones RA, Nicholas B, Mian S, Davies PJ, Griffin M. Reduced expression of tissue transglutaminase in a human endothelial cell line leads to changes in cell spreading, cell adhesion and reduced polymerisation of fibronectin. J Cell Sci 1997; 110: 2461–2472. 9410884 10.1242/jcs.110.19.2461

[bib167] Faye C, Chautard E, Olsen BR, Ricard-Blum S. The first draft of the endostatin interaction network. J Biol Chem 2009; 284: 22041–22047. 19542224 10.1074/jbc.M109.002964 PMC2755928

[bib168] Faye C, Inforzato A, Bignon M, Hartmann DJ, Muller L, Ballut L et al. Transglutaminase-2: a new endostatin partner in the extracellular matrix of endothelial cells. Biochem J 2010; 427: 467–475. 20156196 10.1042/BJ20091594 PMC2876729

[bib169] Wang Z, Perez M, Caja S, Melino G, Johnson TS, Lindfors K et al. A novel extracellular role for tissue transglutaminase in matrix-bound VEGF-mediated angiogenesis. Cell Death Dis 2013; 4: e808. 24052076 10.1038/cddis.2013.318 PMC3789176

[bib170] Ohura N, Yamamoto K, Ichioka S, Sokabe T, Nakatsuka H, Baba A et al. Global analysis of shear stress-responsive genes in vascular endothelial cells. J Atheroscler Thromb 2003; 10: 304–313. 14718748 10.5551/jat.10.304

[bib171] Jones RA, Kotsakis P, Johnson TS, Chau DY, Ali S, Melino G et al. Matrix changes induced by transglutaminase 2 lead to inhibition of angiogenesis and tumor growth. Cell Death Differ 2006; 13: 1442–1453. 16294209 10.1038/sj.cdd.4401816

[bib172] Beckouche N, Bignon M, Lelarge V, Mathivet T, Pichol-Thievend C, Berndt S et al. The interaction of heparan sulfate proteoglycans with endothelial transglutaminase-2 limits VEGF165-induced angiogenesis. Sci Signal 2015; 8: ra70. 26175493 10.1126/scisignal.aaa0963

[bib173] Csomos K, Nemet I, Fesus L, Balajthy Z. Tissue transglutaminase contributes to the all-trans-retinoic acid-induced differentiation syndrome phenotype in the NB4 model of acute promyelocytic leukemia. Blood 2010; 116: 3933–3943. 20739659 10.1182/blood-2010-01-266064

[bib174] Luciani A, Villella VR, Esposito S, Brunetti-Pierri N, Medina D, Settembre C et al. Defective CFTR induces aggresome formation and lung inflammation in cystic fibrosis through ROS-mediated autophagy inhibition. Nat Cell Biol 2010; 12: 863–875. 20711182 10.1038/ncb2090

[bib175] Karpuj MV, Becher MW, Springer JE, Chabas D, Youssef S, Pedotti R et al. Prolonged survival and decreased abnormal movements in transgenic model of Huntington disease, with administration of the transglutaminase inhibitor cystamine. Nat Med 2002; 8: 143–149. 11821898 10.1038/nm0202-143

[bib176] Zhai W, Jeong H, Cui L, Krainc D, Tjian R. *In vitro* analysis of huntingtin-mediated transcriptional repression reveals multiple transcription factor targets. Cell 2005; 123: 1241–1253. 16377565 10.1016/j.cell.2005.10.030

[bib177] D'Souza DR, Wei J, Shao Q, Hebert MD, Subramony SH, Vig PJ. Tissue transglutaminase crosslinks ataxin-1: possible role in SCA1 pathogenesis. Neurosci Lett 2006; 409: 5–9. 17045396 10.1016/j.neulet.2006.08.003 PMC2117902

[bib178] Kahlem P, Green H, Djian P. Transglutaminase action imitates Huntington's disease: selective polymerization of Huntingtin containing expanded polyglutamine. Mol Cell 1998; 1: 595–601. 9660943 10.1016/s1097-2765(00)80059-3

[bib179] Grosso H, Woo JM, Lee KW, Im JY, Masliah E, Junn E et al. Transglutaminase 2 exacerbates alpha-synuclein toxicity in mice and yeast. FASEB J 2014; 28: 4280–4291. 24970392 10.1096/fj.14-251413 PMC4202112

[bib180] Colak G, Johnson GV. Complete transglutaminase 2 ablation results in reduced stroke volumes and astrocytes that exhibit increased survival in response to ischemia. Neurobiol Dis 2012; 45: 1042–1050. 22198379 10.1016/j.nbd.2011.12.023 PMC3276707

